# Underage & Overweight: America’s Childhood Obesity Crisis — What Every Family Needs to Know

**Published:** 2004-09-15

**Affiliations:** University of South Florida, Health Sciences Cente, College of Public Health, Tampa, Fla

*Underage & Overweight: America's Childhood Obesity Crisis — What Every Family Needs to Know* was written primarily for parents, teachers, school administrators, doctors, nurses, other health care professionals, and policymakers. The author, Frances M. Berg, has written a thoroughly researched, evidence-based textbook that emphasizes the importance of a multifaceted approach to alleviating the childhood obesity crisis in the United States. Other books have been written about encouraging healthy lifestyles in children and adolescents, but those authors tend to concentrate on narrowly focused strategies (i.e. diet, exercise, diet and/or exercise) to encourage weight loss among children and adolescents. Anyone who is concerned about, or who has an interest in, the issues and complexities of childhood obesity should obtain a copy of this book and keep it as a reference text.

Berg makes a compelling and convincing argument that "the dangers of childhood obesity are real" by highlighting the increased risks overweight and obese American children face for obesity-related health problems such as type 2 diabetes, hypertension, and psychological disorders. She notes, for example, that from 1979 to 1981, the annual hospital costs related to obesity among children and adolescents were $35 million; from 1997 to 1999, these costs rose to $127 million (1).

Evidence from the literature provides powerful substantiation for Berg's claims about the reality of the dangers of childhood obesity and further supports her hypothesis that American society must take responsibility to "reclaim the health of generations to come." One such published work highlighting the outcomes of the dangers of childhood obesity is a follow-up study to the Harvard Growth Study of 1922 to 1935 by Must et al (2). The results of the study indicate that adolescent overweight is a powerful predictor for a broad range of adverse health effects in adulthood and that these adverse effects are independent of weight in adulthood. Additionally, The National Institute of Diabetes & Digestive & Kidney Diseases reported that the total cost of overweight and obese adults in the United States in 2000 was estimated at $117 billion (3). Sixty-one billion dollars were spent on direct health costs, including preventive, diagnostic, and treatment services, and $56 billion were spent on indirect costs, including income lost by individuals who were unable to work due to morbidity or injury and future income lost due to premature death.

The author has convincingly converted such complicated evidence-based science into language that lay audiences can understand. The book is also well referenced and clarifies the subject matter for readers by including directly into the text definitions of key words, phrases, concepts, and constructs. Readers will not be sidetracked by having to riffle through pages of a glossary or dictionary to find the meanings of unfamiliar words or phrases. Additionally, Berg has provided an excellent resource section filled with practical information relevant to overweight and obesity.

In summary, this is a thoroughly researched and well-referenced textbook on the crisis of childhood obesity in the United States. Berg has taken this complex, multilayered public health problem and reduced it to more understandable units. Berg explains that because so many different factors contribute to the epidemic of childhood obesity, no single solution can alleviate the crisis. To contain this epidemic, the author asserts that all levels of society must advocate healthier lifestyles for healthier children. This book has been written to guide society in achieving this goal.
